# Radiofrequency ablation for treatment of benign thyroid nodules: A PRISMA-compliant systematic review and meta-analysis of outcomes

**DOI:** 10.1097/MD.0000000000004659

**Published:** 2016-08-26

**Authors:** Fen Chen, Guo Tian, Dexing Kong, Liyun Zhong, Tian’an Jiang

**Affiliations:** a Hepatobiliary and Pancreatic Intervention Center; b State Key Laboratory for Diagnosis and Treatment of Infectious Diseases, Collaborative Innovation Center for Diagnosis and Treatment of Infectious Diseases, The First Affiliated Hospital, College of Medicine; c Department of Mathematics; d Department of Ultrasound, The First Affiliated Hospital, College of Medicine, Zhejiang University, Hangzhou, China.

**Keywords:** benign thyroid nodules, meta-analysis, radiofrequency ablation, systematic review, thyroid

## Abstract

**Background:**

Thyroid nodules (TNs) usually appearing in the general population have the potential possibility of malignant transformation and common problems of jugular oppression such as dyspnea and hoarseness. We performed this meta-analysis to evaluate the efficiency of radiofrequency ablation (RFA) for the treatment of benign TNs in accord with the Preferred Reporting Items for Systematic Reviews and Meta-Analyses statements.

**Methods:**

Published literatures were retrieved from PubMed, Embase, Web of Science, and Scopus up to January 27, 2016. Pooled standard mean difference with 95% confidence interval was estimated by fixed- or random-effects model depending on heterogeneity, which was calculated using the Cochran Q, τ^2^, and *I*^2^ statistics. The quality of the articles was evaluated by the Newcastle-Ottawa scale.

**Results:**

Meta-analysis of data from 1090 patients with 1406 benign TNs in 20 articles showed that with the subgroup stratified by nodule volume, they were significantly decreased at 1, 3, 6, 12, and the last follow-up months, when comparing post-RFA with the initial nodule volume. In addition, the volume also notably declined by cold and hot nodules. By subgrouping into the largest diameter, symptom score, cosmetic score, thyrotropin, triiodothyronine, free thyroxine level, and vascularity, the pooled data indicated that there was a decrease in largest diameter, symptom score, cosmetic score, triiodothyronine level, and vascular scale, an unchanged free thyroxine, and an increased thyrotropin level after RFA. The publication bias for this particular study is presented in the following groups: nodule volume reduction at 6 months and last follow-up month after RFA and symptom score.

**Conclusion:**

In summary, by pooling of these studies we recommended that RFA indeed has the advantages in improving outcomes and providing better prognosis for patients with benign TNs.

## Abstract 2

Supplemental Digital Content is available in the text

## 1 Introduction

Thyroid nodules (TNs) are frequent findings, commonly present in 20% to 76% of the general population.^[1]^ Although the majority of TNs are benign, they have the potential possibility of malignant transformation^[2]^ and can pose common problems of jugular oppression such as dyspnea and hoarseness. Thus, these nodules are necessary to be treated.^[3]^ Although previous surgery and levothyroxine therapy are the traditional treatment of choice for benign TNs, both of them have shortcomings in terms of general anesthesia, iatrogenic hypothyroidism, and scar formation.^[4]^ Levothyroxine treatment also showed signs of hyperthyroidism, such as nervousness, palpitations, sweating, or tremor.^[5,6]^ In recent years, image-guided tumor ablations including radiofrequency ablation (RFA), ethanol ablation, and laser ablation (LA) have been clinically and effectively used.^[7–11]^ However, patients who underwent ethanol ablation for TNs complained of voice changes and the direct injury of adjacent nerves or essential structures due to leakage of ethanol.^[12–14]^ It was also reported that there were complications of LA for TNs such as pain in the neck,^[15]^ transient hyperthyroidism,^[16]^ hoarseness,^[17,18]^ and hematoma.^[19]^ For larger nodules, RFA could be performed using the moving-shot technique while LA needs to exit the fiber, risking the possibility of more punctures. Although RFA has been considered to be a safe and effective method of inducing tissue necrosis using thermal energy and has been applied to patients with benign TNs, it may lead to symptoms and cosmetic problems.^[8,20]^ RFA has a risk of inducing autoimmune thyroid disease, recurrent laryngeal nerve palsy, hematoma, skin burns, and adhesion formation if surgery is performed.^[21,22]^ In contrast with surgery, RFA has fewer complications, preservation of thyroid function, and less hospitalization time.^[23]^ A previous meta-analysis observed the diminution in nodule size and improvement of symptom and cosmetic scores after RFA.^[24]^ Nonetheless, emerging reports have focused on the RFA for benign TNs^[4,8,20–23,25–38]^ and since the outcomes including nodule volume, largest diameter, symptom score, cosmetic score, thyrotropin (TSH), triiodothyronine (T3), free thyroxine (fT4) level, and vascularity are still inconclusive, in order to obtain a more precise effectiveness estimation, we conducted a systematic review and meta-analysis.

## 2 Materials and methods

This meta-analysis was conducted on the basis of the Preferred Reporting Items for Systematic Reviews and Meta-Analyses statement.^[39]^ There are no ethical approval and patient written informed consent because of the systematic review and meta-analysis based on the published studies.

### 2.1 Search strategy

To find all relevant publications of RFA for the treatment of benign TNs, electronic searches were independently conducted by 2 individual investigators with the same method in PubMed, Embase, Web of Science, and Scopus databases up to January 27, 2016 using the keywords “radiofrequency ablation,” “RFA,” “RF ablation,” “radiofrequency thermal ablation,” “RTA,” “thyroid,” and “thyroid nodule” (Supplementary Materials). Data were available from the full-published papers and no language or race restriction was used. Bibliographies of relevant review articles were further screened to support the electronic searches.

### 2.2 Inclusion criteria

Included studies have to meet the following criteria: original research papers; prospective or retrospective studies, including cohorts and trials; and clinical results, such as nodule volume, largest diameter, symptom score, cosmetic score, TSH, T3, fT4, and vascularity.

### 2.3 Exclusion criteria

We excluded studies according to the following criteria: abstracts, case reports, case series, in vitro studies, and animal studies were excluded; the studies with malignant TNs were removed; if studies had multiple reports, the latest or most complete article was retained.

### 2.4 Literature screening

Articles were electronically downloaded into reference management software (EndNote X7) and duplicated articles were electronically or manually excluded. The remaining articles were screened by 2 individual investigators using predefined criteria. Full-text versions of potentially relevant articles were available and again screened by 2 individual investigators depending on the predefined criteria. Discrepancy was determined by a third reviewer.

### 2.5 Data extraction and quality assessment

All the information was independently extracted and then cross-checked by 2 investigators according to a standard format as follows: author, publication year, study period, design style, country, population characteristics, treatment methods, number of benign TNs, male or female number, age, follow-up interval, complication, and Newcastle-Ottawa quality assessment (NOS) score. If important data were unavailable in the articles, an email was forwarded to the author for the particular relevant data. Efficacy of RFA for benign TN-related neck symptoms would be estimated by symptom score and cosmetic score. Two types of symptom scores were used: to assess thermal pain thresholds, patients were asked to rate the perceived temperature and pain sensations using numeric rating scales ranging from 0 to 10 (0 indicating “no pain” and 10 being “the maximum pain that can be imagined”)^[21,40]^; the symptoms were listed as follows—pressure symptoms in the neck, difficulty in swallowing (dysphagia), and esthetic complaint. Score (0 = absent; 1 = moderate; 2 = severe) was appraised to each symptom. The sum of these single scores generated a final symptom score ranging from 0 to 6.^[13]^ The physician then estimated a cosmetic score (1, no palpable mass; 2, no cosmetic problem but a palpable mass; 3, cosmetic problem on swallowing only; and 4, easily visible cosmetic problem).^[41]^ Nodule vascularity was graded from 0 to 4 as follows: 0, no signal in the nodule; 1, a few spotty signals in the nodule; 2, signals in <25% of the nodules; 3, signals in 25% to 50% of the nodules; 4, signals in >50% of the nodules.^[42]^ In addition, the quality of each of the included studies was appraised using the NOS scale by 2 reviewers. The NOS scale consists of 8 questions with 9 scores on the basis of 3 parts including selection populations, comparability of groups, and exposure.^[43]^

### 2.6 Statistical analysis

Compared with the initial nodule volume, the meta-analysis checked the changes at 1, 3, 6, 12, and the last follow-up months after RFA. We did the outcome comparison of nodule volume, largest diameter, symptom score, cosmetic score, TSH, T3, fT4 level, and vascularity before the treatment and those at the last follow-up month after RFA treating benign TNs. In addition, we also estimated the subgroup results on the basis of the retrospective and prospective studies. With standard mean difference (SMD) as the effect size, we extracted and combined the mean, standard deviation, and sample size in each study. We transformed and synthesized the indirectly available data to calculate the approximate values based on the previous studies.^[44]^ Heterogeneity within the studies was estimated using the Q statistic,^[45]^ τ^2^, and *I*^2^ (=100% × ([Q − df])/Q).^[46]^ If there was no statistical difference in heterogeneity (*P* ≥ 0.05), the assumption of homogeneity was deemed valid and a fixed-effect model was then applied. Otherwise, a random-effects model would be used. In addition, sensitivity analysis was used to estimate the effect of the remaining studies without the larger one's effect. The risk of publication bias of included studies was checked by the visual inspection of symmetry level of funnel plot and Egger linear regression test.^[47]^ Statistical analysis was implemented by Stata 12.0 software.

## 3 Results

### 3.1 Characteristics of eligible studies in the final analysis

**Figure 1:**
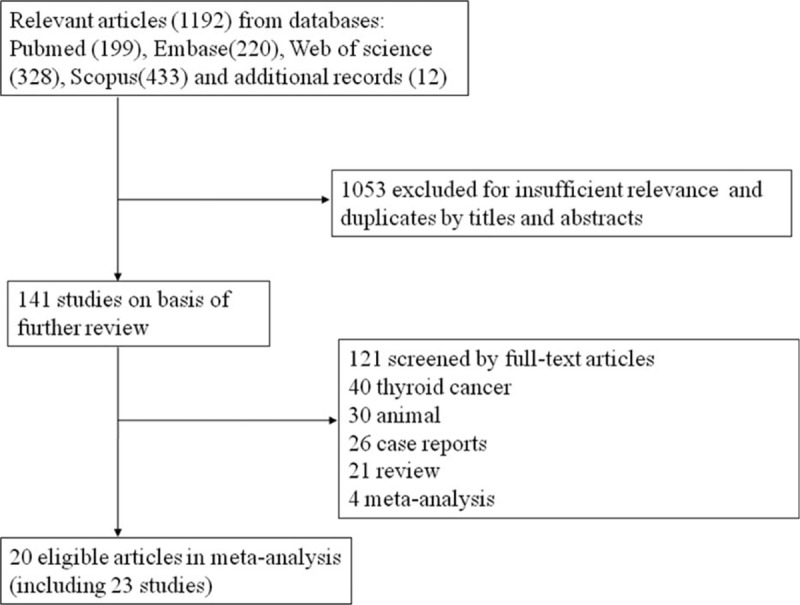
Flow diagram of the study selection process.

A total of 1090 patients with 1406 benign TNs reported in 20 articles were finally identified through the described search strategies till January 27, 2016. One thousand one hundred seventy-two records were removed according to the inclusion criteria (Fig. 1). In these studies reporting age and sex, the age ranges from 13 to 89 years, and 78.62% of the participants were female. The treated nodules in these studies were partly solid, and detailed information on the nodules is shown in population characteristics in Table 1. The follow-up time after RFA is approximately >6 months. The basic characteristics of included studies are listed in Table 1; in addition, the quality of literature evaluated according to NOS scale, which showed good quality with scores of 5 to 8, is listed in Table 2.

**Table 1:**
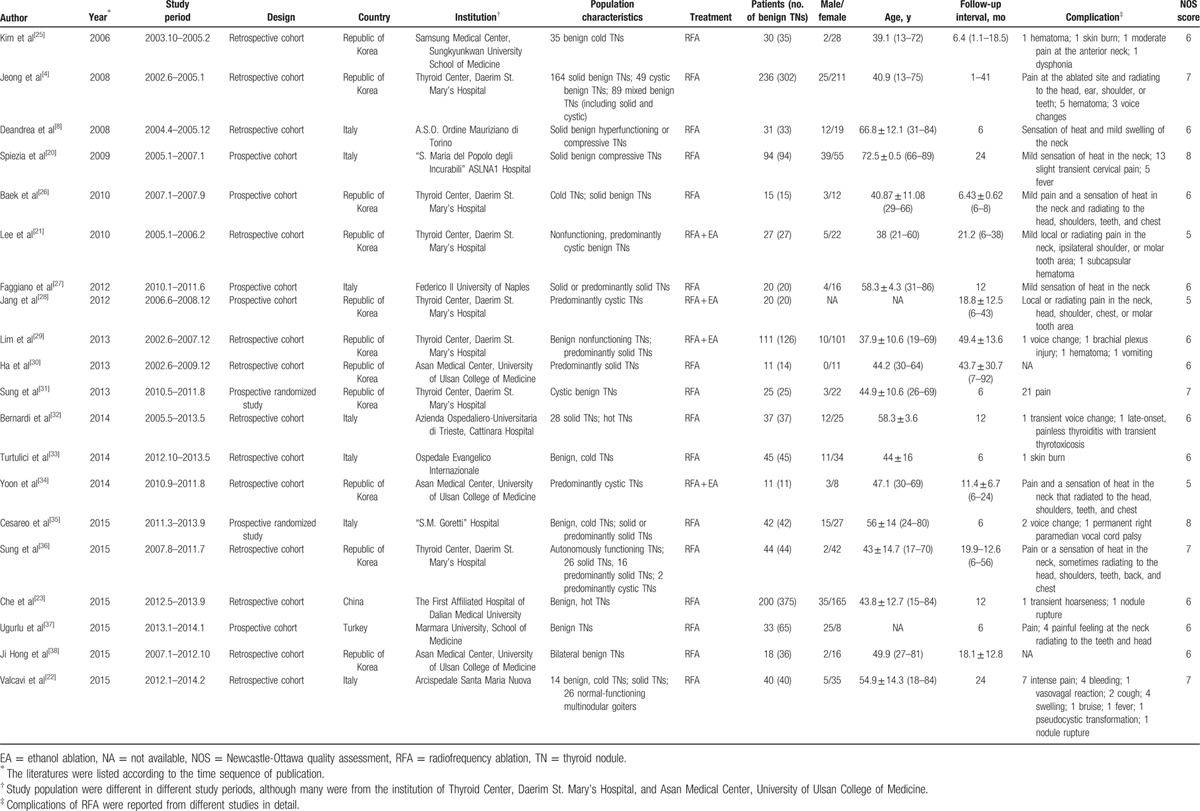
Characteristics of the included studies.

**Table 2:**
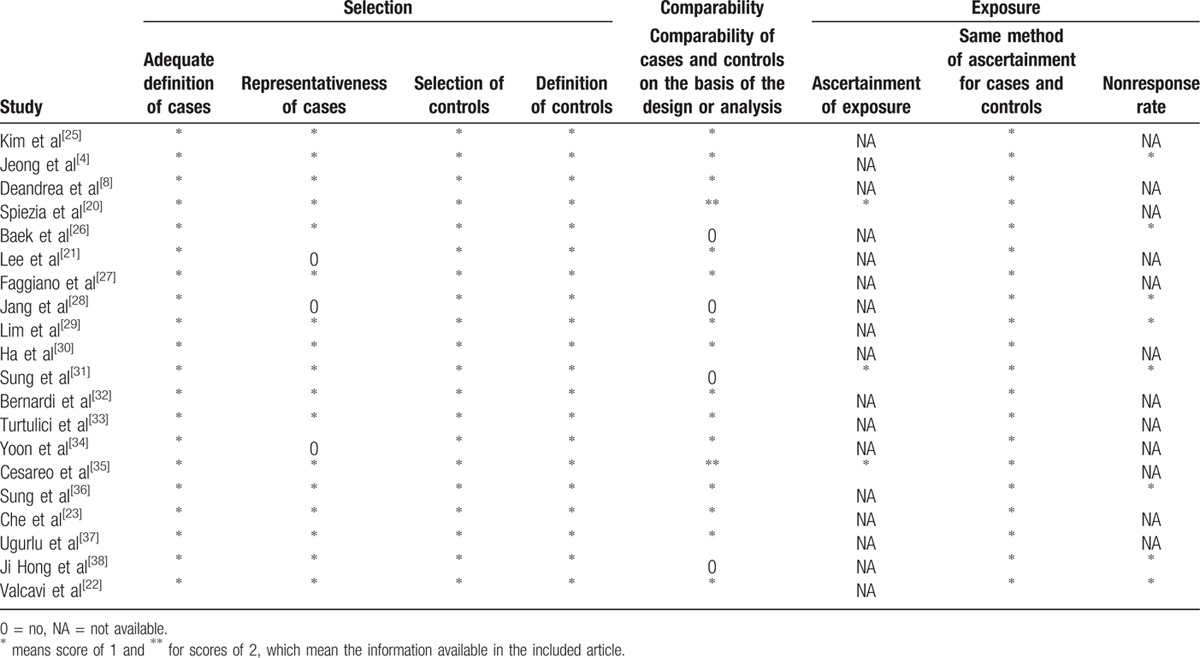
Quality assessment of included studies.

### 3.2 Heterogeneity test result and subgroup analysis

**Figure 2:**
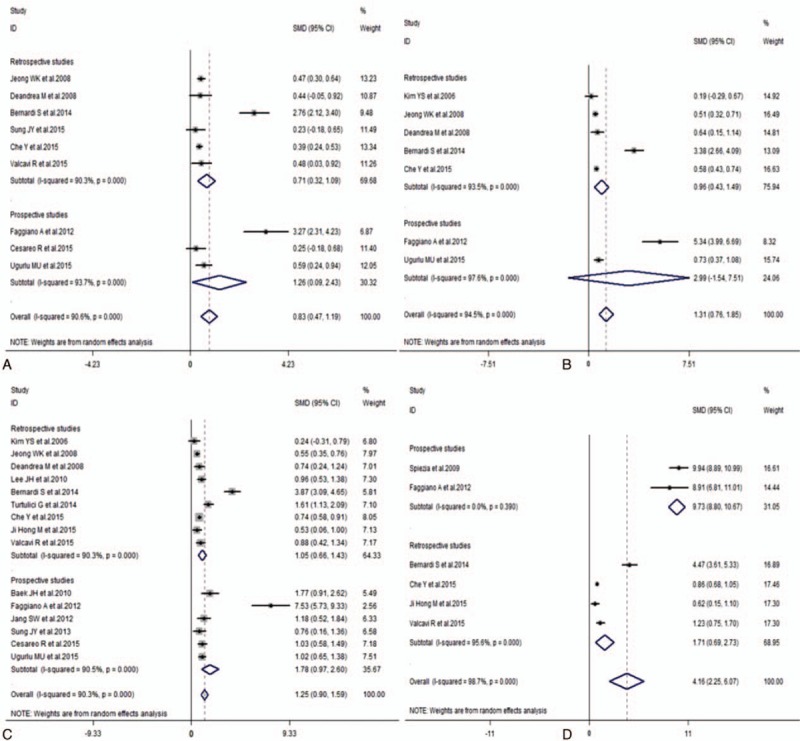
Volume changes of benign TNs before and 1 month (A), 3 months (B), 6 months (C), and 12 months (D) after RFA treatment. Compared with preoperative TN volume, the postoperative volume shows the decreased response at 1, 3, 6, and 12 months after RFA treatment. CI = confidence interval, RFA = radiofrequency ablation, SMD = standardized mean difference, TN = thyroid nodule.

**Figure 3:**
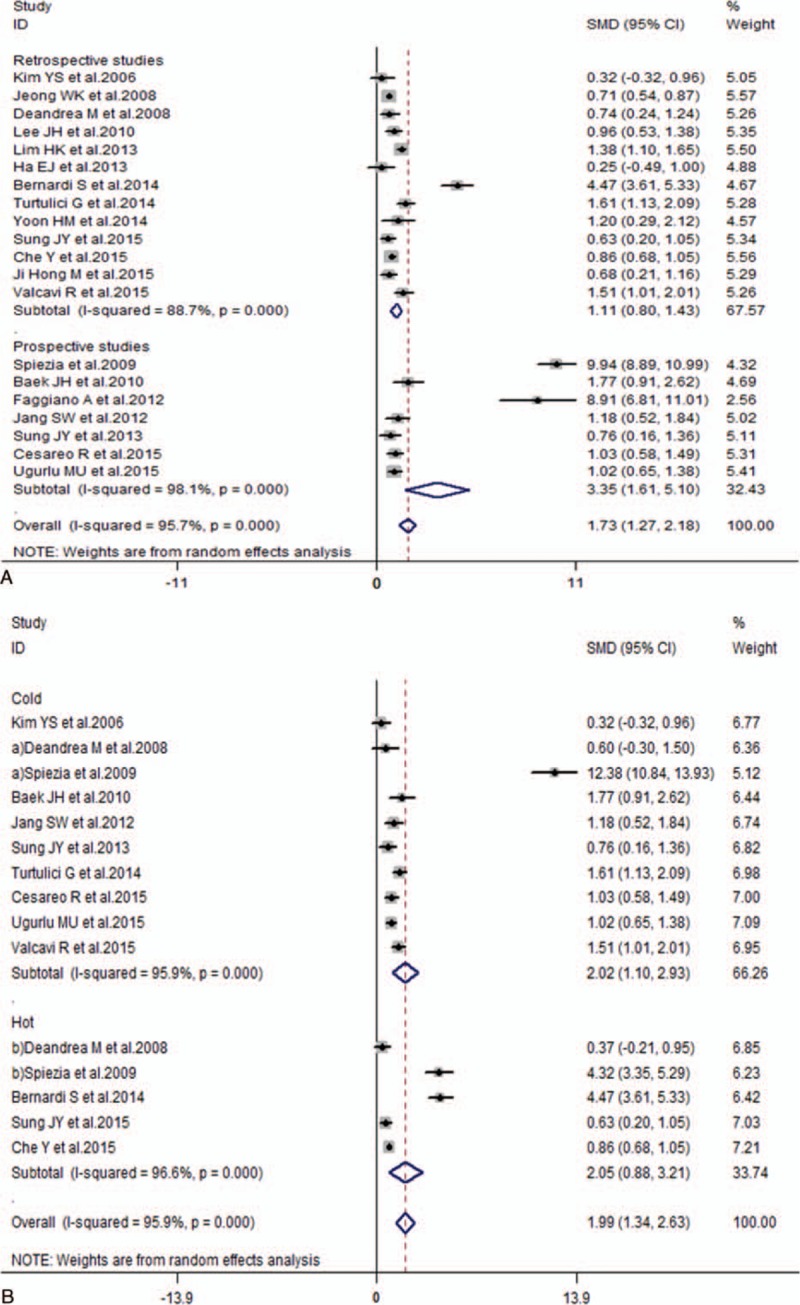
Volume changes of benign TNs before and last follow-up month after RFA treatment. Compared with preoperative TN volume, the postoperative volume indicates the reduced response at the last follow-up month after RFA treatment among overall benign TNs (A) and hot and cold nodules (B). CI = confidence interval, RFA = radiofrequency ablation, SMD = standardized mean difference, TN = thyroid nodule.

With the subgroup stratified by nodule volume, all of the results showed significant decrease at 1, 3, 6, 12, and the last follow-up months after RFA treatment compared to the initial nodule volume (1 month, SMD 95% confidence interval [CI]: 0.83 [0.47–1.19]; 3 months, SMD 95% CI: 1.31 [0.76–1.85]; 6 months, SMD 95% CI: 1.25 [0.90–1.59]; 12 months, SMD 95% CI: 4.16 [2.25–6.07]; last follow-up month, SMD 95% CI: 1.73 [1.27–2.18]) (Table 3; Figs. 2 and 3). In addition, the volume also notably declined by cold and hot nodules (cold nodule, SMD 95% CI: 2.02 [1.10–2.93]; hot nodule, SMD 95% CI: 2.05 [0.88–3.21]) (Table 3). With the subgroup stratified by the largest diameter, symptom score, cosmetic score, TSH, T3, fT4, and vascularity (largest diameter, SMD 95% CI: 1.43 [0.97–1.90]; symptom score, SMD 95% CI: 3.11 [2.28–3.94]; cosmetic score, SMD 95% CI: 2.77 [2.18–3.36]; TSH, SMD 95% CI: −0.44 [−0.86 to −0.02]; T3, SMD 95% CI: 0.33 [0.06–0.60]; fT4, SMD 95% CI: 0.46 [−0.29 to 1.22]; vascularity, SMD 95% CI: 1.78 [0.31–3.25]) (Supplemental Figs. 1–7), the pooled data indicated a decrease in the largest diameter, symptom score, cosmetic score, T3 level, and vascular scale, an unchanged fT4, and an increased TSH level after RFA treatment (Table 3).

**Table 3:**
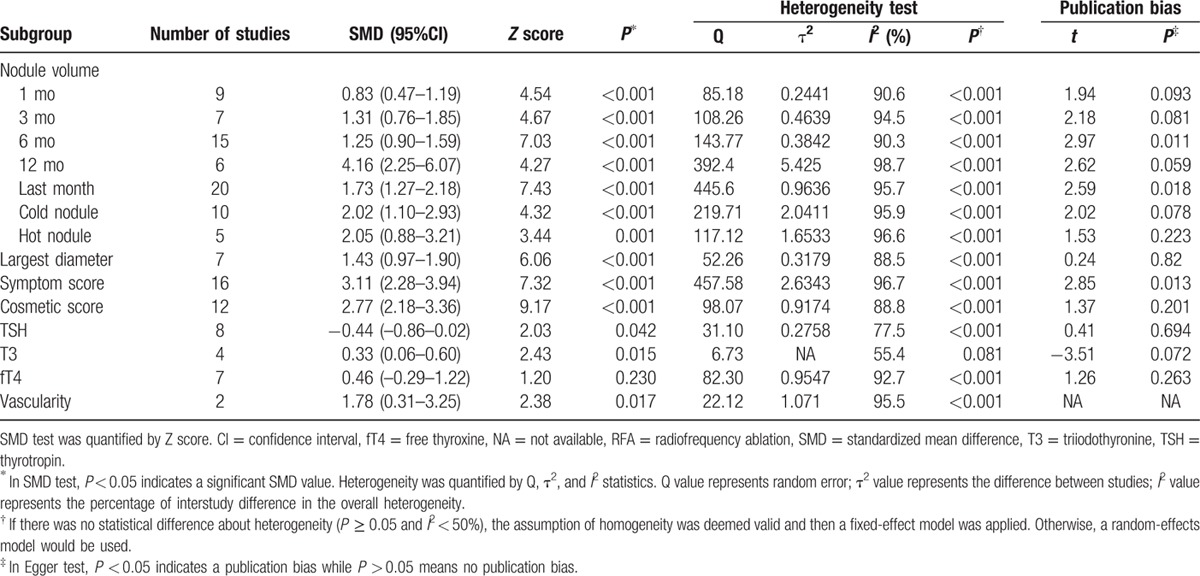
Subgroup analysis of the outcomes before and after RFA.

Additionally, it also suggested similar results for the subgroups on the basis of the retrospective and prospective studies (Figs. 2 and 3; Supplemental Figs. 2–6).

### 3.3 Sensitivity analysis and publication bias

Given the stability of the results, sensitivity analysis was performed and had no significant change if any 1 study was removed. Egger linear regression test was used to appraise the asymmetry of the funnel plot and the risk of publication bias was detected in our meta-analysis as following: nodule volume reduction at 6 months and at the last follow-up month after RFA and symptom score (6 months: *t* = 2.97, *P* = 0.011; last follow-up month: *t* = 2.59, *P* = 0.018; and symptom score: *t* = 2.85, *P* = 0.013) (Table 3).

## 4 Discussion

Nodular thyroid disease is a common finding in endocrine clinical practice, discovered by ultrasound (US) in up to 50% of the general population, with increased prevalence in women and in the elderly. The data of our analysis indicated a decrease in nodule volume, largest diameter, symptom score, cosmetic score, T3 level, and vascular scale, an unchanged fT4, and an increased TSH level after RFA; although some complications of RFA including pain, voice changes, hematoma, and skin burns were reported, RFA yet remains as an effective treatment for patients with benign TNs.

When stratified by nodule volume, all of the pooled results showed significant decrease at 1, 3, 6, 12, and the last follow-up months after ablation. This also occurred in nonfunctioning or autonomously functioning TNs. Single or multiple TNs may develop into autonomously functioning TNs, which can independently induce the production of T3 and T4 without TSH stimulus, suppressing pituitary secretion of TSH and the surrounding normal thyroid function. A study of 236 patients with benign TNs found that the volume of index nodules was reduced from 6.13 ± 9.59 to 1.12 ± 2.92 mL after RFA.^[4]^ Another study also showed that the TN volume decreased significantly from 9.8 ± 8.5 mL before RFA to 0.9 ± 3.3 mL for 4-year follow-up,^[43]^ which was similar to other studies.^[23,36,37]^ Furthermore, the subgroup analysis of largest diameter also supported this noted change. Maybe with even conduction of heat, RFA destroyed the hemorrhagic lesions, directly leading to the thermal degeneration and coagulation necrosis in the cells of the nodules. Although no immune cells were reported in benign TNs after RFA, it showed that 24 months after US-guided PLA of papillary thyroid microcarcinoma, a US-guided fine needle aspiration found the absence of malignant cells, indicating inflammatory cells, charred debris, and fibrous tissue.^[48–50]^ For this reason, we will further explore the RFA treatment of TNs in a future study.

Besides nodule volume and the largest diameter, symptom score and cosmetic score also progressively improved after RFA. Long-term pressure symptoms or cosmetic problems could be relieved, thus suggesting that RFA is a promising way to deal with benign TNs.

Interestingly, the pooled data indicated the reduced T3 level and vascular scale, the unchanged fT4, and the increased TSH level after RFA. Nonetheless, Baek et al observed that after ablation, the serum TSH level in 1 patient improved but still below normal serum hormone levels. This TSH level and clinical scale were not associated with any tumor features or treatment parameters. However, volume reduction was linked with the nodule vascularity of initial US.^[42]^ It was reported that serum TSH, fT4, and T3 levels did not significantly modify from 1.0 ± 0.6 mU/mL, 1.3 ± 0.3 ng/dL, and 152.5 ± 18.6 ng/dL before ablation to 1.3 ± 1.1 mU/mL, 1.3 ± 0.2 ng/dL, and 143.0 ± 16.5 ng/dL, respectively.^[38]^ However, in a multicenter study beyond 19.9-month follow-up, the parameters of fT4, T3, and vascularity markedly decreased from 1.9 ± 1.3, 179.3 ± 102.5, and 3.1 ± 0.7 ng/dL prior to ablation to 1.3 ± 0.4, 133.3 ± 63.1, and 0.9 ± 1.0 ng/dL, respectively, and serum TSH level elevated from 0.12 ± 0.12 uIU/mL prior to ablation to 1.22 ± 0.93 uIU/mL.^[36]^ The physiological fluctuation of these indicators levels may be due to the destruction of the TN and vascularity, and reduced hormones such as T3 and T4, leading to accelerated pituitary secretion of TSH.

There are limitations in our results that should be interpreted. First, the uniformity of the results and between-study heterogeneity could be influenced by the heterogeneity of the inclusion criteria such as sex, age, region, nodule numbers, and nodule size. In this study, we included both retrospective and prospective studies, in which study designs were different, for example, whether diagnostic criteria, test methods, and data acquisition would be uniform. Then we performed subgroup analysis based on the retrospective and prospective studies, and found that the results in limited prospective studies were not inconsistent at 6 follow-up months, for TSH and T3 level before and after RFA treatment. Second, due to the limited sample size, potential confounding factors could reduce the reliability of results. Third, several indirect data transformation methods in the analysis may have an impact on our results. Finally, the included studies were mainly retrieved from Republic of Korea and Italy for RFA treatment of benign TNs, which brought in the selection bias. The possible publication bias in the subgroup could also affect the final outcomes.

Despite these limitations, this study also displayed that RFA might be effective for patients with benign TNs. All published literatures relevant to our issue were retrieved and seriously screened, and data were then extracted in duplicate through protocols. Insufficient data were requested from the authors, and study results were statistically pooled to offer robust estimates of the RFA effectivity.

## 5 Conclusions

In summary, the pooled meta-analysis of included studies demonstrated significant differences in nodule volume, largest diameter, symptom score, cosmetic score, TSH, T3, fT4, and vascularity before and after RFA for patients with benign TNs. RFA has the advantages of improving outcomes and providing better prognosis for patients with benign TNs. Furthermore, to clarify the exact value, more large-scale studies would be undertaken in the future.

## Supplementary Material

Supplemental Digital Content
