# Supplementary material for: Radiofrequency ablation for treatment of benign thyroid nodules: A PRISMA-compliant systematic review and meta-analysis of outcomes
Source: Medicine (Baltimore). 2016 Aug 26;95(34):e4659. doi: 10.1097/MD.0000000000004659 (PMC5400335; doi:10.1097/MD.0000000000004659)
Supplement: Supplemental Digital Content [file medi-95-e4659-s001.doc]

**Search strategy:**

Pubmed (1950-present)

1. ("RFA" OR "radiofrequency ablation" OR "RF ablation" OR "radiofrequency thermal ablation" OR "RTA")
2. Thyroid
3. 1 AND 2
4. "Ablation Techniques"[Mesh]
5. "Catheter Ablation"[Mesh]
6. "Thyroid Nodule"[Mesh]
7. 4 OR 5
8. 6 AND 7
9. 3 OR 8

Embase(1980-present)

1. 'radiofrequency ablation':ab,ti
2. 'rfa':ab,ti
3. 'rf ablation':ab,ti
4. 'radiofrequency thermal ablation':ab,ti
5. 'rta':ab,ti
6. 1 OR 2 OR 3 OR 4 OR 5
7. thyroid:ab,ti
8. 6 AND 7

Scoups

1. TITLE-ABS-KEY ( “radiofrequency ablation” )
2. TITLE-ABS-KEY ( "RTA" )
3. TITLE-ABS-KEY ("RF ablation" )
4. TITLE-ABS-KEY ("radiofrequency thermal ablation" )
5. TITLE-ABS-KEY ("RTA" )
6. 1 OR 2 OR 3 OR 4 OR 5
7. TITLE-ABS-KEY ("thyroid" )
8. 6 AND 7

Web of science

1. TS=(ablation)
2. TS=(thyroid nodule)
3. 1 AND 2
4. TI=( ablation)
5. TI=(thyroid nodule)
6. 4 AND 5
7. 3 OR 6
